# Children’s stress influences their diet, physical activity and adiposity: longitudinal behavioural and hormonal pathways

**DOI:** 10.1186/2049-3258-72-S1-O1

**Published:** 2014-06-06

**Authors:** Nathalie Michels, Isabelle Sioen, Liesbet Boone, Caroline Braet, Els Clays, Inge Huybrechts, Barbara Vanaelst, Stefaan De Henauw

**Affiliations:** 1 Department of Public Health, Ghent University, Ghent, Belgium; 2 Department of Developmental, Personality and Social Psychology, Ghent University, Ghent Belgium

## Background

Psychosocial stress and adiposity are important public health threats that have been associated with each other. Longitudinal studies are needed to reveal the directionality and underlying behavioural and hormonal factors. In young children, literature is scarce. We aim to study the longitudinal associations of children’s stress with lifestyle and adiposity.

## Materials and methods

In 312 Belgian children (5-12y) of the ChiBS study, the two-year longitudinal stress-lifestyle-adiposity relation was examined. Stress data (sum score of negative events, problem behaviour and negative emotions), lifestyle (food consumption, psychological eating behaviour, physical activity by accelerometry, screen time, sleep duration) and adiposity (BMI, fat% by BodPod, waist) were measured. At baseline, also salivary cortisol levels (4 samples, 2 days) were determined as a biomarker for stress. Multilevel time modelling examined the cross-sectional relation of cortisol with diet. Mplus cross-lagged analyses tested the longitudinal stress-lifestyle-adiposity relations and moderation.

## Results

Children with a high stress score reported more sweet food consumption, psychological eating behaviour (emotional eating, external eating, restrained eating) and physical activity. Some of these relations were sex- and age-dependent. No effects on sleep duration were found. The stress effect on adiposity was depending on moderators. Sweet food consumption and cortisol were enhancing moderators: stress increased adiposity in children with high sweet food consumption (BMI, p=0.020) or high cortisol awakening response (waist, p=0.030). Physical activity was a protective moderator: stress decreased adiposity in children with high physical activity (fat%, p=0.025). In the other direction, high BMI (p<0.001), high fat% (p<0.001) and psychological eating behaviour (p=0.014) also increased stress. High cortisol (overall levels, awakening response and diurnal slope) was associated with an unhealthy diet especially with the sweet foods.

## Conclusions

The association of cortisol with diet supports the theory of cortisol–induced comfort food preference. Indeed, children’s stress deteriorates their diet which stimulates adiposity. On the other hand, stress can also enhance physical activity which inhibits adiposity. This creates a perspective for multi-factorial obesity prevention, targeting stress and lifestyle factors in parallel. Concerning lifestyle, the environment should be an ‘activity encouraging, healthy food zone’ that minimizes opportunities for stress-induced eating. Concerning stress, appropriate stress coping skills should be acquired. Moreover, psychological support for obese children should be organized.

